# Thyroid surgery in children: a single-center experience of 20 years

**DOI:** 10.1590/1806-9282.20240001

**Published:** 2024-07-19

**Authors:** Kutay Bahadir, Selin Ural, Javid Abdullayev, Abdurrahman Karaman, Mesut Parlak, Adil Boz, Gungor Karaguzel

**Affiliations:** 1 Akdeniz University, School of Medicine, Department of Pediatric Surgery – Antalya, Turkey.; 2 Akdeniz University, School of Medicine, Department of Pediatric Endocrinology – Antalya, Turkey.; 3 Akdeniz University, School of Medicine, Department of Nuclear Medicine – Antalya, Turkey.

**Keywords:** Hypocalcemia, Pediatrics, Thyroid gland, Thyroid nodule, Thyroidectomy

## Abstract

**OBJECTIVE:**

Thyroidectomy is a relatively uncommon procedure in pediatric patients. We aimed to review our 20-year experience of thyroid surgery.

**METHODS:**

A total of 39 patients who underwent thyroid surgery from 2003 to 2023 were retrospectively evaluated. All patients were followed preoperatively and postoperatively by our institutional multidisciplinary board. Patients were divided into two groups based on their pathologies: benign and malignant.

**RESULTS:**

In total, 39 patients (27 girls and 12 boys) underwent 47 thyroid surgeries (total thyroidectomy in 19 patients and subtotal thyroidectomy in 20 patients, with 8 of them having completion thyroidectomy). Notably, 20 (51%) patients had benign and 19 (49%) patients had malignant pathologies. Median age at operation was 157 (9–223) months in the benign group and 182 (1–213) months in the malignant group. In the benign group, 12 (60%) patients had colloidal goiter and 8 (40%) patients had other conditions. In the malignant group, 12 (63%) patients had papillary thyroid carcinoma, 3 (16%) patients had follicular thyroid carcinoma, 2 (11%) had medullary thyroid carcinoma, and 2 patients had other thyroid malignancies. Overall permanent complication rate was 2 out of 39 (5%), which was similar for both groups (1 hypocalcemia in each group). The median follow-up was 38 months (1–179 months) with no local recurrence or distant metastasis.

**CONCLUSION:**

Pediatric thyroidectomies are performed on a heterogeneous group of pediatric patients due to a diverse group of pathologies. A multidisciplinary approach is required for proper initial management and surgical strategy with decreased complication rate and event-free survival of these patients in experienced tertiary centers.

## INTRODUCTION

Thyroidectomies are performed in approximately 50,000 cases annually, but only 500 of these procedures are performed in children^1^. Even though thyroidectomy is one of the most commonly performed surgeries, it is still relatively uncommon in children^1-4^. Pediatric thyroidectomy is indicated for various conditions such as goiter that causes compressive symptoms, Graves’ disease, thyroid nodules, thyroid cancer, and prophylaxis in familial endocrine syndromes such as multiple endocrine neoplasm 2^2,5,6^. Thyroid nodules have an incidence of 1–2% in the pediatric population, which is less common than in adults^6-8^. However, the malignancy rate is 22–26% and is more likely in the pediatric population, with papillary thyroid carcinoma (PTC) (80–90%) accounting for the majority, followed by follicular thyroid carcinoma (FTC) (10%) and medullary thyroid carcinoma (MTC) (3–5%) and rarely less differentiated malignancies of the thyroid^6-9^. This results in a more aggressive approach in children than in adults^6,8^. Even though thyroid cancer in children is rare and accounts for less than 1% of all childhood cancers, overall malignancy rates are increasing throughout the years^5,6,10-13^. The reported risk factors for malignancy include autoimmune disorders such as Hashimoto's thyroiditis and Graves’ disease, iodine deficiency, and radiation exposure^9^.

Physical examination, radiological imaging, and pathological evaluations can be utilized for the differential diagnosis of thyroid nodules^9^. Thyroid nodules are usually present as an asymptomatic neck mass, cervical lymphadenopathy, or compression symptoms may accompany^9^. On ultrasonographic imaging, irregular shape and margins, microcalcifications, marked hypoechogenicity, intranodular hypervascularization, enlargement of the nodule over time, invasion of extra thyroid tissues, anteroposterior diameter larger than the transverse diameter, and the presence of cervical lymphadenopathies suggest malignancy^9,14^. Ultrasound-guided fine needle aspiration (FNA) biopsy can be performed on hypofunctioning thyroid nodules, and histopathological evaluation according to the Bethesda classification can be evaluated to predict the risk of malignancy^9^.

In the literature, complications related to thyroidectomies have a higher incidence in children than adults, mainly due to less experience in the pediatric population^2,4^. These complications include hematoma, hypoparathyroidism, hypocalcemia, vocal cord paralysis, nerve injury and vascular, and tracheal or esophageal injury^2-4^.

The aim of this study is to review 20 years of pediatric thyroidectomy experience with a multidisciplinary approach in a single institution and to compare complication rates between malignant and benign thyroid disease.

## METHODS

### Study design and data collection

A total of 39 pediatric patients (age below 18 years) who underwent thyroid surgery in a single tertiary center from 2003 to 2023 were evaluated retrospectively by an institutional multidisciplinary board. All procedures performed in this study were in accordance with the ethical standards of the institutional and national research committee and with the Helsinki Declaration and its later amendments or comparable ethical standards. This study was approved by our institutional ethics committee (KAEK-454). Written consent was obtained from the patient's parents or legal guardians prior to surgery.

Data were collected from the Akdeniz University School of Medicine, Department of Pediatric Surgery and Pediatric Endocrinology. Gender, age at operation, concordant diseases, number of operations performed and surgical approaches, pathological results, and postoperative complications were noted. Vascular sealing devices and nerve monitoring were used selectively. All patients were followed preoperatively and postoperatively by our institutional multidisciplinary board. Patients were divided into two groups according to benign and malignant pathologies. The follow-up duration of each patient was noted.

### Statistical analysis

Descriptive statistics were calculated for all variables. The association between benign and malignant disease with specific outcomes, including hypocalcemia and vocal cord paralysis, was analyzed using Fisher's exact probability test using SPSS for Windows (SPSS 23.0; IBM, Armonk, NY, USAC). The level of statistical significance was set to p<0.05.

## RESULTS

A total of 39 patients underwent 47 thyroid surgeries, of whom 27 were girls and 12 were boys. Of note, 19 children had a total thyroidectomy, and 20 children had a subtotal thyroidectomy. Eight children who underwent subtotal thyroidectomy primarily had a completion thyroidectomy after pathological results. In addition, 20 (51%) patients had benign and 19 (49%) patients had malignant pathologies (6 of them underwent modified neck dissection) (Table 1). The median age at operation was 157 (9–223) months for benign pathologies and 182 (1–213) months for malignant pathologies. In the benign group, 12 (60%) patients had colloidal goiter and 8 (40%) had other conditions. In the malignant group, 12 (63%) patients had PTC, 3 (16%) had FTC, 2 (11%) patients had MTC, and 2 patients had other thyroid malignancies. Five children had prophylactic total thyroidectomy due to a RET oncogene mutation.

**Table 1:** Number of benign and malignant thyroid disease.

Diagnosis		Number of patients (n=39)
Benign (goiter)		20
	Colloidal hyperplasia/goiter	12
	Dishormogenetic goiter	3
Benign (other conditions)		5
	Hashimoto's thyroiditis	3
	Normal thyroid (thyroid lobectomy for parathyroid hyperplasia)	1
	Thymus (intrathyroidal)	1
Malignant		19
	Papillary thyroid carcinoma	12
	Follicular thyroid carcinoma	3
	Medullary thyroid carcinoma	2
	Hurtle cell carcinoma	1
	Immature teratoma	1

Preoperatively, FNA was performed with ultrasound guidance on 25 patients (64.1%) who presented with thyroid nodules. Notably, 8 of these children (32%) had nondiagnostic unsatisfactory results, while 9 (36%) had benign and 8 (32%) had malignant results. Two children with nondiagnostic FNA results were diagnosed with malignant conditions after thyroidectomy.

Hypocalcemia was significantly more frequent in the malignant group (p<0.05). The overall permanent complication rate was 2 out of 39 (5%), which was similar for both groups (one hypocalcemia in each group). Three patients who underwent total or completion thyroidectomy along with modified neck lymph node dissection had intensive care unit admission due to vocal cord paralysis (p=0.106). One of these patients had a tracheostomy while in the intensive care unit. All the vocal cord paralysis was transient and the tracheostomy was decannulated before discharge. The recurrent laryngeal nerve injury was not seen in either group. The overall rate of complications was higher for operations for malignancy (p<0.01) (Table 2). Radioactive iodine therapy was applied to 13 patients. The median follow-up period was 38 months (1 month– 179 months). There was no local recurrence or distant metastasis.

**Table 2:** Number of complications in benign and malignant thyroid disease groups.

Complications		Benign (n=20)	Malign (n=19)
Hypocalcemia (n=8)			
	Transient	0	6
	Permanent	1	1
Vocal cord paralysis (n=3)			
	Transient	0	3
	Permanent	0	0

## DISCUSSION

Thyroidectomy is a relatively uncommon procedure in the pediatric population and is indicated for hyperthyroidism, thyroid nodules, malignancy, goiter with compressive symptoms, and prophylaxis in children with familial endocrine syndromes that cause a predisposition to thyroid malignancies^1-4^. This study aims to compare complication rates between benign and malignant thyroid diseases in pediatric patients with a multidisciplinary approach in a single institution.

Benign thyroid diseases in children are mainly Graves’ disease, chronic autoimmune thyroiditis (Hashimoto's thyroiditis), colloid goiter, and thyroid adenomas^15^. Benign thyroid pathologies can be managed with anti-thyroid medication. However, if these treatments fail, surgical management or radioactive iodine treatment is an alternate viable option^6,16^. The literature shows that radioactive iodine treatment in children can increase secondary malignancy rates, which causes concern, especially in children younger than 5 years of age^16^. In this study, 20 patients (51%) underwent thyroidectomy for benign thyroid pathologies. One of the patients who had a thyroidectomy for a benign pathology had permanent hypocalcemia. Hypocalcemia reportedly presents up to 20% in children following thyroidectomies, 8% of which can become permanent^3^. Young patient age, hyperthyroidism, and lymph node dissection are known risk factors for hypocalcemia^3^.

Thyroid cancer in children is rare and accounts for less than 1% of all childhood cancers^6^. Thyroid nodules are also less common in children and are not necessarily malignant but have a higher rate of malignancy compared to adults^6,8^. Due to this increased malignancy risk of nodules in children, FNA is recommended to be performed under ultrasonography guidance^7^. Studies show that about 35% of FNA results were indeterminate^7^. In our study, 32% of the FNA results were indeterminate and 25% of them were malignant after operation.

Radiation, iodine deficiency, and genetic predisposition syndromes are known risk factors for thyroid carcinoma. Children especially have thyroid tissue that is more vulnerable to radiation, and Hashimoto's thyroiditis increases the risk of malignancy of a thyroid nodule^6^. Furthermore, overall malignancy rates are increasing throughout the years both for adults and children^5,6,10-13^. Pediatric thyroid malignancies are mostly well-differentiated thyroid carcinomas (DTC), accounting for 0.5–3% of all childhood cancers^6,13,17^. PTC is the most common type of DTC in children, accounting for about 60–80% of all pediatric DTCs; FTC and MTC are less common^6,13^. In children, PTC is more aggressive, and local and regional involvement at the time of diagnosis is around 60%, which is more common at diagnosis than in adults^5,6,18^. In the literature, the most common pathology in pediatric thyroidectomy cases is PTC, followed by FTC and Graves’ disease^1^. In this study, 12 patients (31%) underwent thyroidectomy due to PTC. Five of these patients who had undergone thyroid lobectomy due to benign or inconclusive FNA results underwent completion thyroidectomy. Chen et al. argued that the rate of lymph node metastasis is about 60–80%^19^. All 12 patients underwent neck lymph node sampling at the time of operation, and 6 of these patients were positive for carcinoma and underwent modified neck lymph node dissection. Modified neck lymph node dissection and total thyroidectomy are known risk factors for hypoparathyroidism^1^. In our series, persistent hypoparathyroidism developed in two patients.

Follicular thyroid carcinoma and MTC account for less than 10% of all pediatric thyroid malignancies^6,20,21^. In this study, three cases were diagnosed as FTC accounted for 16%. MTC in children is usually familial and related to RET oncogene mutations and syndromes such as MEN 2A and 2B^6^. Prophylactic thyroidectomy at different ages is recommended based on the risk stratification of these patients^6^. In our series, five patients underwent prophylactic thyroidectomy based on the RET mutation, and two of these patients had MTC on pathological evaluation. On the contrary, one patient who underwent total thyroidectomy for a degenerative thyroid nodule was diagnosed with Hurtle cell carcinoma.

Thyroid teratomas are rare and generally benign pathologies^3^. However, immature teratomas have been reported rarely in literature^3^. Generally, this malignancy rate increases with age^3^. In our series, one patient underwent total thyroidectomy due to a neonatal immature teratoma without any complications postoperatively.

Postoperative complications of thyroidectomy are most commonly hypocalcemia due to trauma or devascularization of parathyroid glands^7^. According to the literature, 35.5% transient and 4.2% permanent hypocalcemia occur following thyroidectomy in children^7^. Permanent hypocalcemia is associated with central or lateral neck dissection^22^. In our series, transient and permanent hypocalcemia complications occurred in 15 and 5.1% of patients, respectively. In our series, hypocalcemia, both transient and permanent, was related to lymph node dissection. Both the patients who developed permanent hypocalcemia had undergone lateral lymph node dissection. Another possible complication of thyroidectomy is laryngeal nerve injury, which can be unilateral or bilateral. Unilateral injury results in hoarseness, choking, and aspiration of food or water, while bilateral injury can be a life-threatening condition blocking the airway^7^. The literature shows that recurrent laryngeal nerve injury rates are higher in children than in adults^16^. In our series, three patients with PTC, who underwent total or completion thyroidectomy along with modified neck lymph node dissection, developed bilateral vocal cord paralysis, which was resolved in follow-up. In recent years, the use of nerve stimulation to protect the laryngeal nerve has become a standard approach. We presume that the rates of laryngeal nerve injury will decrease in the following years.

In the tertiary center where this study was conducted, all patients were evaluated by the pediatric endocrinology, pediatric surgery, and radiology departments prior to surgery to form a treatment plan. The medical treatment of the patients was planned by the pediatric endocrinology department. Ultrasonographic evaluation and FNA were performed by the radiology department. All patients who required thyroid scintigraphy were referred to the nuclear medicine department. All of the operations were performed by the pediatric surgery department after a multidisciplinary meeting of all the departments involved in the evaluation of the patient.

This study has several limitations. First of all, this is a retrospective study, which may cause data-related bias. Although the single-center design of this study allows for a more standard protocol of surgery procedure and follow-up, it also accounts for another limitation of the small sample size.

## CONCLUSION

Pediatric thyroidectomies are performed on a heterogeneous group of pediatric patients due to a diverse group of pathologies. Higher recurrent laryngeal nerve injury and hypocalcemia rates in thyroidectomies have been associated with low-volume centers, especially in the pediatric population. This study supports that a multidisciplinary approach is crucial for the proper initial management and surgical strategy and can decrease complication rates and event-free survival of these patients in experienced tertiary centers.

## ETHICAL APPROVAL

Written informed consent was given by the patients parents/legal guardians. This study was approved on 07/06/2023 by the İnstitutional Ethical Committee (KAEK- 454). The study was carried out in accordance with Decleration of Helsinki.

## ABBREVIATIONS:

PTC: papillary thyroid carcinoma
FTC: follicular thyroid carcinoma
MTC: medullary thyroid carcinoma
MEN2: multiple endocrine neoplasm
FNA: fine needle aspiration
DTC: differentiated thyroid carcinomas
